# Population Size in Evolutionary Biology Is More Than the Effective Size

**DOI:** 10.1111/eva.70029

**Published:** 2024-10-22

**Authors:** Joachim Mergeay

**Affiliations:** ^1^ Research Institute for Nature and Forest Geraardsbergen Belgium; ^2^ Ecology, Evolution and Biodiversity Conservation KU Leuven Leuven Belgium

**Keywords:** conservation genetics, diversity, evolutionary potential, information theory, theoretical population genetics

## Abstract

In population genetics idealized Wright‐Fisher (WF) populations are generally considered equivalent to real populations with regard to the major evolutionary processes that influence genotype and allele frequencies. As a result we often model the response of populations by focusing on the effective size *N*_*e*_. The Diversity Partitioning Theorem (DPT) shows that you cannot model the behavior of a system solely on the basis of a diversity (accounting for unevenness among items) without taking richness into account. I show that the census population size (the number of adults, *N*_*c*_) is equivalent to a richness, and that the effective size *N*_*e*_ is equivalent to a true diversity. It follows logically from the DPT that we require both *N*_*e*_ and *N*_*c*_ to understand how drift, selection, mutation, and gene flow interact to shape the course of evolution of populations. Here I review evidence that both *N*_*c*_ and *N*_*e*_ affect evolutionary trajectories of populations for neutral and adaptive processes. This also influences how we should consider evolutionary potential and genetic criteria for conservation of populations. The effective size of a population is of huge importance in evolutionary biology, but it should not be the sole focus when population size is concerned. Applied evolutionary studies need to integrate *N*_*c*_ in the equation more consistently when modeling the response to selection, mutation, migration, and drift.

## 1 Introduction

“Imagine observing a set of genotype frequencies in a biological population. It would then be natural to construct an idealized population using theory that approximates the biological population. This is an attempt to construct an idealized population that is equivalent to the actual population from the perspective of the processes influencing genotype frequencies” (Hamilton 2009).

In population genetics, we often define the effective size *N*_*e*_ of a real population on the basis of how much genetic drift it experiences compared to an ideal Wright‐Fisher (WF) population. The size of the WF population experiencing the same amount of drift is then the effective size of that population (Wright 1931). A population of *N*_*c*_ = 1000 individuals may experience the genetic drift of a WF population of *N*_*e*_ = *N*_*c*_ = 100 individuals. This makes theoretical modeling of the behavior of populations far less complex. However, we often assume that this simplification hardly matters to other fundamental evolutionary processes: mutation, migration, and selection (Hamilton 2009). To better understand the theoretical basis of why and when the WF model may be adequate or not, it helps to understand that *N*_*e*_ is a summary statistic of a population, and consider population size in light of entropy and diversity (Jost 2006).

First, I show how we can understand the rather abstract concept of effective population at a much more intuitive level once we realize the relation between census size and effective size is conceptually related to how the number of alleles and heterozygosity are complementary measures of genetic diversity. Next, I provide different examples of how and when the evolutionary impact of mutation and selection changes when *N*_*c*_ differs from *N*_*e*_. Far from being a comprehensive review, it is mostly intended to stir a debate on how we can improve our take on the effective population size for better conservation of populations, and integrate other aspects of population size in population genetics, when needed.

### 1.1 Richness and Diversity, Numbers and Effective Numbers

Diversity indices express the number of different elements in a system, which we can express through Hill numbers (Hill 1973; Jost 2006). We often think of the number and diversity of species, but it applies equally to different alleles. Richness, a diversity index of order zero (Hill 1973), counts the number of different elements in the system and is insensitive to the frequency of each element. Diversity indices of higher orders (1: Shannon entropy; 2: Gini‐Simpson index, …) become increasingly sensitive to the unevenness among elements. It is important to distinguish between a diversity index (which has no units and varies from 0 to 1), and the deduced true diversity, which is expressed as an effective number or the numbers equivalent of a diversity index (Jost 2006). These Hill numbers can be used to partition in a standardized way biological diversity of all kinds: within and among populations (genetic), among species and regions (community ecology and phylogeny), and across multiple spatial levels (Gaggiotti et al. 2018). Using this framework, richness can be decomposed into independent elements of evenness (scaling from 0 to 1) and a diversity or effective number of any desired order (Jost 2010) as D0=Dq/E0,q, with D0 the diversity of order zero (the richness) and Dq the diversity of order *q*. As the evenness $E_{0,q}$ (of orders 0 and *q*) among elements approaches unity (complete evenness), the true diversity approaches the richness value. The consequence of this Diversity Partitioning Theorem (DPT) is that richness is an essential aspect of any diversity index: We cannot model the properties of a system solely by its evenness‐based diversity (Jost 2010). Generalizing, the effective number represents how many different elements there are in an equivalent idealized system where the evenness is maximized and the diversity equals the richness (Jost 2006).

Two commonly used variables in population genetics are the number of alleles (A_*n*_ or *n*_*a*_, the diversity of order zero), and the expected heterozygosity *H*_*e*_ (Nei 1987). Note that in the literature the term allelic richness (AR or *A*_R_) is often used for the number of alleles in a sample of standardized size, and allelic diversity is used to refer to the absolute number of alleles in a population (e.g., Caballero and García‐Dorado 2013). Here I will further consider richness as the diversity of order zero, and define allelic richness as the actual number of alleles in a population (*n*_*a*_), with *n*_*e*_ used to define the effective number of alleles (Crow and Kimura 1970). For both *n*_*a*_ and *H*_*e*_, there is a sound theoretical framework that explains what values to expect given a certain effective size *N*_*e*_ and the mutation rate *μ* (Wright 1931; Kimura and Crow 1964; Crow and Kimura 1970; Ewens 1972; Nei, Chakraborty, and Fuerst 1976; Nei and Tajima 1981). *H*_*e*_ is the Gini‐Simpson index, the diversity index of order 2 (Jost 2006), expressed as $H_{e}=1-\sum p_{i}^{2}$, with *p*_*i*_ the frequency of the *i*th allele (Nei 1987). From *H*_*e*_ we can deduce the effective number of alleles as $n_{e}=1/\left(1-H_{e}\right)$ (Jost 2006), which we can now compare with the allelic richness *n*_*a*_ to provide the evenness $E=n_{e}/n_{a}$. Table 1 provides an example that illustrates the dependence of the effective number of alleles *n*_*e*_ and gene diversity *H*_*e*_ on allelic richness *n*_*a*_, as expected from the DPT. It also shows the complementary nature of the information deduced from both types of summary statistics. In Table 1, Pop1 and Pop3 are ideal populations in terms of allele frequency distributions for the studied locus (evenness = 1 and *n*_*e*_ *= n*_*a*_). Pop2 and Pop3 have *n*_*e*_ = 2.0, but population 2 has higher *n*_*a*_. Focusing only on *H*_*e*_ or *n*_*e*_, we would consider these populations to be identical. If this locus were under selection, however, Pop2 would have higher evolutionary potential owing to higher *n*_*a*_ (Caballero and García‐Dorado 2013). Pop4 has the same *n*_*a*_ as Pop1 and Pop2, but has the lowest *n*_*e*_ and is most prone to inbreeding.

**TABLE 1:** Allelic richness (*n*_*a*_), gene diversity (*H*_*e*_), evenness $E=n_{e}/n_{a}$ and the effective number of alleles (*n*_*e*_) for four populations (Pop1—Pop4), at a single locus with five alleles A–E.

Allele	Pop1	Pop2	Pop3	Pop4
A	0.20	0.16	0.50	0.01
B	0.20	0.10	0.50	0.01
C	0.20	0.04	0.00	0.01
D	0.20	0.02	0.00	0.01
E	0.20	0.68	0.00	0.96
*n*_*a*_	5	5	2	5
*H*_*e*_	0.800	0.500	0.500	0.078
Evenness	1.00	0.40	1.00	0.22
*n*_*e*_	5.00	2.00	2.00	1.08

Similarly, we can express census and effective sizes of stable haploid populations with discrete generations in a form that satisfies the DPT (D0=D2/E0,2) as $N_{c}=N_{e}V_{k}$ (Hill 1979) with *N*_*c*_ the number of adults in the population (the population size richness) and *V*_k_ the demographic variance in reproductive success. Since the reciprocal of the variance is an evenness of order 2 (Hill 1997), it logically follows that *N*_*e*_ is a diversity of order two, and that *N*_*e*_ alone cannot suffice to describe a population's size, nor its response to evolutionary processes. Hill (1979) originally considered *N* as the population size of a cohort of newborns, with *V*_*k*_ being determined by lifetime variance in reproductive success from cradle to cradle, but we can equally describe this for adults, considering *N*_*c*_ as the census size, and *V*_*k*_ the corresponding variance from adults to the next generation of adults, which leads to exactly the same outcome for *N*_*e*_ (Waples 2024).

For diploid monoecious or dioecious sexual populations, the variance of interest is slightly different, as it includes terms for Mendelian segregation, environmental stochasticity and differences among sexes (Engen, Lande, and Saether 2005), but it generally satisfies the form of the equation D0=Dq/E0,q that the effective size depends on the census size in the numerator and the variance in reproductive success in the denominator. Kimura and Crow (1963) express this dependency of *N*_*e*_ on *N*_*c*_ as a second‐order term with the inbreeding $N_{e}=\frac{4N_{c}-2}{V_{k}+2}$ in diploid monoecious stable populations. For more complex situations, additional variants are deduced from these general expressions, including corrections for overlapping generations (Hill 1972, 1979), for fluctuating population size, and separate sexes (Kimura and Crow 1963), but the general tenet remains: The census size is a richness (the number of potential breeders without taking differences in reproductive output into account) and the effective size is a diversity of order 2 (the effective number of breeders). Mark that in the DPT, the effective number can never exceed the richness. In diploid organisms, we have the particular situation where *N*_*e*_ equals *N*_*c*_ when$\;\bar{k}=V_{k}=2$ and reaches its maximum value at *V*_*k*_ = 0, but it never exceeds the number of genomes in the population 2*N*_*c*_, a few exceptions aside (Wang and Hill 1999; Santiago and Caballero 2000).

Since effective population size is a true diversity, it follows from the DPT that we cannot correctly model the behavior of populations solely on the basis of *N*_*e*_; we also need information on *N*_*c*_. Below I highlight some important consequences.

## 2 When *N_e_* Differs From *N*_*c*_: Implications for Evolutionary Processes

In recent decades, there has been an increasing recognition that most problems in biology are not strictly ecological or evolutionary in nature, and they are increasingly described as eco‐evolutionary dynamics (Pelletier, Garant, and Hendry 2009). Whereas evolutionary processes depend (at least partially) on *N*_*e*_, ecological processes (competition, predation, population growth rates, epidemiology, etc.) depend on the census size *N*_*c*_ and the reproductive capacity *K*. However, there are complex contemporary feedbacks between ecological and evolutionary processes, necessitating a better integration in population genetics practices. The actual units on which everything acts (ecology) are individuals, not their effective equivalents. It is therefore important to be able to decouple *N*_*e*_ from *N*_*c*_. In the WF model of population genetics, however, they are linked 1:1.

Allendorf et al. (2024; this issue) provide a clear demonstration of this need for decoupling, by showing that the number of alleles can vary as a function of *N*_*c*_ at a constant *N*_*e*_, with cascading effects on the long‐term evolutionary potential of populations (see also Caballero and García‐Dorado 2013).

Kimura and Ohta (1969a) derived the time to extinction of a neutral mutant (or migrant) allele *i* in a population as

(1) $$T_{\text{loss}}=-4N_{e}\mathrm{PLn}\left(P\right)/\left(1-P\right)$$

with *P* the frequency of allele *i*. We can also deduce from this the time to fixation if we substitute *P* by (*1*−*P*). This equation is sometimes used to indicate that fixation time scales with *N*_*e*_ (Kliman, Sheehy, and Schultz 2008), but this is neither entirely wrong nor complete. If we consider the number of allele copies of *i* to be *a*, we can rewrite $P=a/\left(2N_{c}\right)$ and substitute it in Equation 1:

$$T_{\text{loss}}=-4N_{e}\frac{a}{2N_{c}}\mathrm{Ln}\left(\frac{a}{2N_{c}}\right)/\left(1-\frac{a}{2N_{c}}\right)$$

This shows that the time we can expect a particular neutrally behaving allele copy to linger depends both on the effective size *N*_*e*_ and the census size *N*_*c*_ of the population (Table 2). Of course, this merely shifts the problem to determining the frequency of the allele. For abundant alleles, typical sample sizes in population genetic studies are sufficient to estimate the frequency and approximate *T*_loss_ without the need to know *N*_*c*_, but it becomes interesting when we consider single allele copies arisen through mutation. In a sample of individuals aimed to represent the population, we don't notice this frequency difference, but it is far from negligible once selection is involved. A neutral mutant or immigrant allele arising in an ideal population with *N*_*e*_ = *N*_*c*_ = 100 is expected to be lost after 18.6 generations, whereas in a more realistic setting where *N*_*c*_ would be 10 times higher for the same *N*_*e*_ (*N*_*c*_ = 1000), that same allele is expected to be lost after only 2.8 generations. Admittedly, since *N*_*c*_ is 10 times larger in this example, the number of alleles arising through mutation will on average also be 10 times larger, but these will typically be different alleles. If the *N*_*e*_/*N*_*c*_ ratio is even smaller, the difference is further exacerbated (Table 2). In other words, the fixation probability of a new allele depends on *N*_*c*_, but the time to fixation on *N*_*e*_. Even though this was already noted by Crow and Kimura (1970), they dismissed this difference as hardly relevant because the *N*_*e*_ to *N*_*c*_ ratios they assumed were much larger (0.9) than what we typically see today (average: 0.10, range 0.5–10^−5^) (Hoban et al. 2020).

**TABLE 2:** Expected time to extinction (number of generations) of an allele copy as a function of *N*_*e*_ (leftmost column) and the *N*_*e*_ */N*_*c*_ ratio (top row). As *N*_*e*_ */N*_*c*_ decreases, the time to extinction of a singleton allele converges to zero no matter how large or small the *N*_*e*_.

*N*_*e*_	*N*_*e*_ */N*_*c*_ ratio			
	1.0	0.1	0.01	0.001
10^1^	10.23	1.86	0.28	0.04
10^2^	18.61	2.77	0.37	0.05
10^3^	27.66	3.68	0.46	0.06
10^4^	36.85	4.61	0.55	0.06
10^5^	46.05	5.53	0.64	0.07
10^6^	55.26	6.45	0.74	0.08
10^7^	64.47	7.37	0.83	0.09
10^8^	73.68	8.29	0.92	0.10
10^9^	82.89	9.21	1.01	0.11

When we calculate the time to loss of a neutral singleton allele for a given *N*_*e*_ and for various *N*_*e*_ */N*_*c*_ ratios, we see that for a large part of the parameter space the time to extinction of a singleton allele nearly only depends on the *N*_*e*_ */N*_*c*_ ratio, and hardly on *N*_*e*_ itself (Table 2). Mark that for low *N*_*e*_ */N*_*c*_ ratios, the expected time to extinction is nearly instantaneous (within 1 generation), irrespective of the *N*_*e*_. This may have consequences for how we evaluate the role of gene flow relative to dispersal, or for that matter to mutation, but also for the way nearly neutral and conditionally neutral alleles affect evolutionary potential.

Mutation is thought to mostly lead to deleterious or neutral alleles, and only a small proportion is thought to yield beneficial alleles (Fisher 1930). But it is crucial to realize that they occur in the census population. When *N*_*c*_ = 10*N*_*e*_, there are 10 times more mutation events in the focal population than when *N*_c_ = *N*_*e*_. Hence, the probability that a beneficial allele arises is also 10 times larger than what we typically expect. If behaving neutrally, it gets eliminated on average also much faster (Equation 1, Table 2). When selection comes into play, however, the fixation probability can change dramatically (see below).

### 2.1 Selection Acts on Individuals

When we use the WF model to predict evolutionary responses of populations to selection, we assume the census size equals the effective size, or that this simplification has a negligible influence on the population trajectory. However, the actual targets of selection are the different individuals, which reflect the population size richness. This distinction matters for two reasons: First, sexual recombination and mutation can create a sheer infinite number of possible genotypes, each with different genotype‐by‐environment interactions. When *N*_*c*_ = 10*N*_*e*_ the population has 10 times more tickets in the lottery of life than in a WF population, and the efficiency of selection will be higher too, as also the tails of the distribution of genotypic and phenotypic values (on which selection acts) will be longer. This general dependency of the efficiency of selection on *N*_*c*_ has been well established for decades (Caballero 2020), yet its influence in species with very small *N*_*e*_/*N*_*c*_ remains much less known, especially to what extent it affects the maintenance of evolutionary potential. Second, even when assuming the number and diversity of genotypes is the same (e.g., 100 genotypes, represented by 10 or 1 individuals each), the probability that an intrinsically better‐adapted genotype is killed by bad fortune is smaller when *N*_*c*_ = 1000 than in an ideal population where *N*_*e*_ = *N*_*c*_ = 100. “The fittest fish stands no earthly chance if its lake dries out” (Sigmund 1993). The above example is a crude simplification, as it assumes that all drawn lottery tickets are independent, within and among families. In reality, there may be genetic, spatial, and ecological reasons for correlated survival within families (Crow and Morton 1955; Waples and Anderson 2017), which can affect the outcome of the interaction between drift and selection differently.

### 2.2 There's More Than One Population Size

When we consider the relation between the richness and the diversity of a population, we typically think of the census size and the effective size, with the census size the number of potential parents in a population (Waples 2022). Allendorf, Hössjer, and Ryman (2024) built on this difference to showcase how the number of alleles varies as a function of *N*_*c*_ for fixed values of *N*_*e*_. In ideal populations survival of zygotes to adulthood is random, leading to *k* = *V*_*k*_ = 2 in stable populations. However, selection acts on every life stage during the life cycle of each organism, leading to non‐random patterns of survival. As fecundity increases (more zygotes are produced for a fixed *N*_*c*_ and *N*_*e*_) the scope for survival being less and less random increases (Caballero 2020). Lifetime fecundity per individual varies enormously across species: Some species have a maximum lifetime fecundity inferior to 10 (e.g., black rhinoceros, Nhleko, Parker, and Druce 2017), whereas explosive breeders may have lifetime fecundity exceeding 30 million offspring (e.g., Atlantic cod, Jobling and Pedersen 1995). Also long‐lived trees, corals, fungi, algae, orchids, and many other species across the tree of life are capable of producing millions of offspring per individual. This yields a census size for the adult cohort (the number of reproducing individuals in the population, typically considered “the census size” *N*_*c*_), but also for the totality of the population including immature life stages (*N*_max_). These differences in population size across life stages become especially important in species with very large fecundity values, yielding extreme differences between *N*_max_ and *N*_*e*_.

Fecundity (also known as reproductive capacity *K*) has been shown to be strongly positively correlated with neutral genome‐wide diversity (Romiguier et al. 2014), and therefore long‐term effective population size. Complementary to that, the efficiency of selection (which depends on both *N*_*e*_ and *N*_*c*_; Caballero 2020) and the recombination rate (which scales with *N*_*e*_) also affect genome‐wide diversity patterns through linked selection or genetic draft (Corbett‐Detig, Hartl, and Sackton 2015; Ellegren and Galtier 2016). Genetic draft is the reduction of genetic diversity at loci physically linked to loci under selection (Gillespie 2000), a process that can be very important during selective sweeps. As a result, the genome‐wide genetic diversity of large populations may be much more affected by genetic draft than by genetic drift (Ellegren and Galtier 2016). Although genetic draft and genetic drift are very different processes, when we look at effective size in highly fecund species from a conservation perspective, we should acknowledge that the *N*_*e*_ (short term and long term) may be affected by other factors than genetic drift, thereby altering our expectations for effects of inbreeding and adaptative potential in such species when we consider their *N*_*e*_ values (Santiago and Caballero 2016; Charlesworth and Jensen 2021).

### 2.3 The Number of Alleles in a Population

Ewens (1972) derived that the expected number of alleles in a population at mutation‐drift equilibrium can be expressed as

(2) $${\hat{n}}_{a}=\frac{\theta }{\theta }+\frac{\theta }{\theta +1}+\frac{\theta }{\theta +2}+\ldots +\frac{\theta }{\theta +2N-1}$$

with $\theta =4N_{e}\mu$, *N*_*e*_ the effective size, *N* the population size in the parent population, and *μ* the mutation rate. Mark that “population size” is rather ambiguously defined, as it may reflect both *N*_max_ and *N*_*c*_ (the number of adults). If we consider a semelparous species with discrete generations, the number of alleles decreases as the cohort matures, and there is no single equilibrium value; rather there is a stable oscillation between the maximum *n*_*a*_ just after reproduction and the minimum *n*_*a*_ just prior to reproduction. Typically, the parent cohort included more individuals (having reproductive success *k* = 0) than those that reached adulthood; some of those that reached adulthood may not have reproduced, and those that reproduced may have failed to produce offspring that survived to adulthood. At the end of their lives, the evolutionary contribution of each of these individuals with *k* = 0 is the same, and it doesn't matter whether they died as juveniles or as senescent adults. For a sample of size *n*, we can substitute in equation 3 *N* by *n* to obtain the expected allelic richness of the sample. Also Crow and Kimura (1970 eq. 9.6.13, p. 455) highlight that the number of alleles depends on both *N*_*e*_ and *N*_*c*_. The consequences of this relation have recently been highlighted by Allendorf, Hössjer, and Ryman (2024), showing that the (neutral) number of alleles in a population depends on both *N*_*e*_ and *N*_*c*_, with differences becoming increasingly larger as the *N*_*e*_ */N*_*c*_ ratio decreases.

Note that Ewens' formula depends on unrealistic assumptions of mutation‐drift equilibrium, neutrality, and independent segregation of loci. Here it is merely used to illustrate the dependency of *n*_*a*_ on *N* (whether *N*_*c*_ or *N*_max_), and it is intended to show the reader that the difference can be pronounced. Now consider a population of size *N*_*e*_ and a genome‐wide nucleotide mutation rate *μ* = 10^−8^ per generation (Lynch 2010). With an average length of 10^4^ nucleotides of a functional locus (the average size of a gene under potential selection) (Strachan and Read 2019), this yields a locus‐wide mutation rate of 10^−4^. The expected gene diversity at equilibrium is given by ${\hat{H}}_{e}=\frac{\theta }{\theta +1}$ with θ = *4N*_*e*_ *μ* (Wright 1931). Given a certain *N*_*e*_, we can now calculate ${\hat{n}}_{a}$ and ${\hat{H}}_{e}$ for a WF population and for actual populations with different *N*_*c*_. Table 3 provides an example of the difference in neutral allelic richness caused by ignoring the role of *N*_*c*_ for common values of *N*_*e*_, *N*_*c*_, and *H*_*e*_. It shows that true allelic richness in typical situations is underestimated by 7% to >100%, even if that difference is not measurable in a typical sample. In extreme cases (*N*_*e*_/*N*_max_ = 10^−8^, which is realistic in explosive breeders that have millions of offspring per individual), the true *n*_*a*_ of the population can be orders of magnitude larger than what we assume by only focusing on *N*_*e*_. This extra allelic richness hardly affects the estimated *n*_*a*_ of a typical biological sample, because the alleles are extremely rare. However, this has important implications for long‐term evolutionary potential (see below).

**TABLE 3:** Population‐level expected allelic richness $\hat{n_{a}}$ (derived from Equation 2) and related summary statistics at mutation‐drift equilibrium in a typical locus for effective sizes of 10^2^, 10^3^, and 10^4^ at *N*_*e*_ */N*_*c*_ ratios of 1, 0.1, and 0.01. A nucleotide mutation rate = 10^−8^ and a locus size = 10^4^ nucleotides would yield a net average locus‐wide mutation rate of μ = 10^−4^. For illustration purposes, I also provide $\hat{n_{a}}$ for a sample size of 30, and the gene diversity *H*_*e*_. Neither are measurably affected by different *N*_*e*_ */N*_*c*_ ratios. As *N*_*c*_ increases for a stable *N*_*e*,_ *N*_*e*_ */N*_*c*_ obviously decreases, and $\hat{n_{a}}$ increases. This effect becomes more and more pronounced as the effective population becomes larger.

*N*_*e*_	100	1000	10,000
*ϴ*	0.04	0.4	4
*H*_*e*_	0.04	0.29	0.80
*n*_*e*_	1.04	1.40	5.00
*n*_*a*_ (*N*_*e*_ */N*_*c*_ = 1)	1.23	4.06	34.59
*n*_*a*_ (*N*_*e*_ */N*_*c*_ = 0.1)	1.32	4.99	43.80
*n*_*a*_ (*N*_*e*_ */N*_*c*_ = 0.01)	1.42	5.91	54.01
*n*_*a*_ (*n* = 30)	1.16	2.40	9.14

To illustrate the role of *N*_max_ in allelic richness, consider species where *N*_max_ exceeds the reciprocal of the mutation rate by several orders of magnitude. For example, a female Atlantic cod (*Gadus morhua*) produces on average half a million eggs per kg of body mass (Jobling and Pedersen 1995). For a modest adult census size of 5 million individuals, the actual number of individuals of all stages subjected to selection easily amounts to 10^13^ individuals. Each of these individuals represents a unique genotype on which selection acts in permanence. Assuming a germline mutation rate of 10^−8^ mutations per nucleotide per genome per generation and a genome size of 900 Mb (Hardie and Hebert 2004), this yields for this population a total expected amount of 10^14^ new nuclear germline mutations every year. Although the bulk of these alleles will be lost by drift and bad fortune in the blink of an eye every generation, there is an incredible allelic richness and genotypic richness on which selection can act in that population: Every year reproduction gives rise to 10 trillion genetically unique new targets of selection, housing a huge array of different alleles that originates from scratch every generation. On average, every nucleotide position in the genome is variable in such a population just after reproduction. Natural selection in such populations must be extremely efficient, compared to similar sized *N*_*e*_ populations of species that have at most a dozen offspring per individual over a lifetime (e.g., large mammals). An individual's chance of ever winning the lottery is extremely small, and yet every week there is a winner. It just requires enough participants (*N*_max_). This raises the question to what extent in populations of explosive breeders long‐term evolutionary potential is influenced by *N*_*c*_ and *N*_max_, rather than by *N*_*e*_ and genetic drift?

### 2.4 Evolutionary Potential Also Depends on *n_a_* and *N*_*c*_

The fate of non‐neutral alleles in a population is typically approximated by use of a diffusion equation (Crow and Kimura 1970; Charlesworth 2009). This allows us to deduce that the probability of fixation of a newly arisen allele depends on the selection coefficient *s*, the effective size (determining the rate of drift), and the census size (determining the number of mutant alleles occurring each generation). This is shown (for a biallelic autosomal locus with semi‐dominance) by

$$Q\approx \frac{N_{e}s}{N}\;\frac{1}{(1-\exp\left(-2N_{e}s\right)}$$

As long as the value of $2N_{e}s$ falls between −1 and 1, the allele tends to behave neutrally because genetic drift is so pervasive relative to selection (Charlesworth 2009). When it is larger than 1, the allele's chance to become fixed increases rapidly. For neutral alleles the probability of fixation is entirely dependent on their frequency.

However, Schuster and Sigmund (1989) showed that the diffusion approximation can sometimes vastly underestimate the probability of fixation of beneficial alleles. They showed that, as fecundity *N*_max_ increases, beneficial alleles will have fixation probabilities that are nearly independent of *N*_*e*_ and on their initial frequency. According to their results, any advantageous allele that survives the first few rounds of genetic drift and manages to occur in a few copies tends to get picked up by natural selection and persists in the population. Hence the fixation probability mostly depends on the fitness advantage provided, and much less on the frequency (2N^−1^). Again, this feeds back to the role of *N*_max_ and the actual number of alleles *n*_*a*_ in the population, which we know depends on *N*_*e*_ and *N*. Since the frequency of the beneficial allele is of marginal importance (Schuster and Sigmund 1989), having a larger *N*_max_ leads to a larger *n*_*a*_ and a higher chance of having a beneficial allele in the population, and therefore a higher evolutionary potential (Caballero and García‐Dorado 2013). As *N*_max_ can be orders of magnitude larger than *N*_*e*_ in explosive breeders, its importance could be large. And yet, as the absolute frequency of new mutant alleles decreases, their stochastic elimination from the population is also expected to occur very rapidly (Table 2). In summary, there is a gap in our understanding of this interaction between selection and drift when *N*_*e*_ and *N*_max_ differ strongly.

**FIGURE 1:**
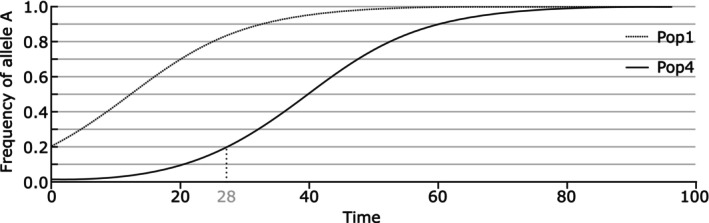
Evolutionary potential depends on richness. Imagine population 1 and population 4 from Table 1 being confronted with a new environment in which there is directional selection for one of the alleles. Assuming semi‐dominant fitness effects, the time to 90% fixation for allele A would be 32 time units in Pop1, and 60 time units in Pop4 (the number of generations depends on the absolute fitness difference, but expressed as relative time units the exact fitness difference is irrelevant). On average over all alleles, the average time to 90% fixation is in this case 1.45 times as long for population 4 (starting from *p* = 0.01 in four cases and *p* = 0.96 in one case) relative to population 1 (starting from *p* = 0.20 in five cases). The shape of the curve for an allele trajectory from 0.20 to 0.90 is identical to that from 0.01 to 0.90, except for the head start of 28 time units it has from 0.01 to 0.20. The evolutionary potential (the likelihood of reaching the end point), however, is identical as long as no alleles are lost by drift.

High gene diversity (*H*_*e*_) is considered to cause high evolutionary potential because it is positively correlated with additive genetic variance (Frankham et al. 1999). This is in part a correlation that is a consequence of the richness and the evenness that cause high gene diversity (Table 1): When many alleles are present (high allelic richness) and they have an even frequency distribution (high allelic diversity and therefore high *H*_*e*_), the average distance to target of any beneficial allele under directional selection is simply shorter (Figure 1). The time to fixation from an allele frequency 0.01 to 1.00 is shorter than from 0.20 to 1.00 due to the head start of the latter. True, the absolute rate of fitness change depends on the additive genetic variance (Fisher 1930) and the target will be reached faster. The raw evolutionary potential (Figure 1), however, does not depend on the distance to target, and is also a function of the allelic richness of loci under selection. This was also shown earlier by Caballero and García‐Dorado (2013), showing that short‐term evolutionary potential is primarily affected by diversity, and long‐term evolutionary potential by allelic richness. Allendorf, Hössjer, and Ryman (2024) also showed that populations with larger *N*_*c*_ have higher allelic richness, and therefore the evolutionary potential in these populations is also higher than in corresponding WF populations with the same *N*_*e*_.

## 3 Neutral Genetic Diversity Matters for Conservation

It has been argued that genome‐wide (neutral) genetic diversity should not be the main focus of conservation efforts because there is no simple relationship between genetic diversity and extinction risk (Teixeira and Huber 2021). This argument was effectively countered on several instances (e.g., DeWoody et al. 2021; García‐Dorado and Caballero 2021; Kardos et al. 2021), highlighting that higher levels of genome‐wide genetic diversity are associated with higher levels of additive genetic variance (which allows for rapid evolutionary responses), lower drift load and a lower risk of mutational meltdown.

Moreover, many loci are conditionally neutral (Anderson et al. 2013). A locus could be truly under mild selection but behave neutrally when 1 > 2*N*_*e*_s > −1 (Charlesworth 2009). Genotype‐by‐environment interactions cause some alleles to behave neutrally or selectively, depending on the environment. Under changing conditions such alleles can in principle shift from mildly deleterious/neutral to beneficial very rapidly. When we consider evolutionary potential under changing conditions, the fate of (conditionally) neutral alleles and the expected duration of segregation (Kimura and Ohta 1969b) are of crucial importance.

Neutral, nearly neutral, and beneficial alleles behave differently with respect to *N*_*e*_ and *N*_*c*_. Frequencies of neutral alleles are largely determined by drift (ignoring draft), and hence *N*_*e*_. Since mildly deleterious alleles often behave nearly neutrally and since these alleles make up an important part of the genetic load (Kimura, Maruyama, and Crow 1963), the risk of a mutational meltdown (Lynch, Conery, and Burger 1995), will be primarily affected by *N*_*e*_. Simulations by Kardos et al. (2021) included a fixed *N*_*e*_ */N*_*c*_ ratio of 0.25 to demonstrate the role of mutational meltdown in relation to genome‐wide genetic diversity and *N*_*e*_. And yet, some species like the water flea *Daphnia* seem to escape mutational meltdown in spite of being governed by strong founder effects (Louette et al. 2007; Haileselasie et al. 2018) and low *N*_*e*_ (Vanoverbeke and De Meester 2010). Furthermore, they manage to maintain evolutionary potential following bottlenecks (Chaturvedi et al. 2021). They do have extremely large *N*_*c*_ and hence extremely small *N*_*e*_/*N*_*c*_, however, which suggests a non‐negligible role for *N*_*c*_ in evolutionary potential (Allendorf, Hössjer, and Ryman 2024).

There are many species with very large *N*_max_ in relation to typical values of *N*_*e*_, and many are of management or conservation concern, such as trees, corals, orchids, macro‐algae, but also commercially exploited fish and crustacean species, … Many explosive breeders are found among invasive species that pose threats to native biota. A better understanding of to what extent evolutionary potential and the minimum viable population size (MVP) are affected by the interplay between *N*_*e*_ and *N*_*c*_ or *N*_max_, and genetic diversity versus allelic richness seems non‐trivial in some parts of the parameter space. Similarly, to what extent populations can cope with deleterious load and/or are at risk of extinction seems to be affected by reproductive rates: Simulations by Pérez‐Pereira et al. (2022) indicated that populations with high reproductive rates (the number of offspring *K*, which ties to *N*_max_) are more likely to survive a bottleneck than species with low values of *K*. The parameter space they explored was reflective of species with relatively small reproductive rates (*K* < 60), and generally reflected the need for effective population sizes ranging in the hundreds (high *K*) to more than 1000 (low *K*). It would be highly relevant to conservation to expand such analyses to populations with very high reproductive rates (*K* reaching thousands to millions).

### 3.1 Consequences for Conservation

A heated debate arose in the 1980s and 1990s on the minimum size a population should have to avoid inbreeding depression and perpetually retain evolutionary potential. Threshold estimates varied tenfold between 500 < *N*_*e*_ < 5000 (Franklin 1980; Soulé 1980; Lande 1995; Frankham and Franklin 1998; Franklin and Frankham 1998; Lynch and Lande 1998), but have settled meanwhile between 500 < *N*_*e*_ < 1000 (Frankham, Bradshaw, and Brook 2014). Despite pronounced disagreements between both sides, the influence of richness parameters (*n*_*a*_, *N*_*c*_, and *N*_max_) on evolutionary potential has largely been ignored. Although these thresholds seem to be robust (Traill, Bradshaw, and Brook 2007; Pérez‐Pereira et al. 2022), there is still a lot we don't know.

Many efforts are now focused on estimating *N*_*e*_ for conservation purposes (Hoban et al. 2020, 2022), and in some species and situations, this is rather more challenging than at first glance (Waples 2016; Ryman, Laikre, and Hössjer 2019). My deductions indicate that for some parts of the parameter space, *N*_*c*_ may be crucial to understand the evolutionary potential of populations. Since maintenance of this adaptive capacity is at the heart of current efforts to preserve and monitor genetic diversity (Mastretta‐Yanes et al. 2024), it seems crucial to grasp more fully how *N*_c_ and *N*_*e*_ interact to shape evolutionary potential across different life histories.

In general, it seems that richness statistics (*n*_*a*_, *N*_*c*_, *N*_max_) can influence evolutionary trajectories, and this may impact estimates of the MVP size. High fecundity can decrease a population's extinction risk and reduce MVP thresholds (Pérez‐Pereira et al. 2022). Having high reproductive rates *K* is akin to a situation where *N*_max_ *> > N*_*e*_, although there is a clear need to further explore the influence of values of *K* and *N*_max_ that are several orders of magnitude larger than *N*_*e*_ on population survival and threshold values for conservation. When *N*_max_ is very large, populations of explosive breeders (with very high *K*) might be less dependent on high *N*_*e*_ to maintain evolutionary potential. Integrating *N*_*c*_ and *N*_max_ (or fecundity *K*) into conservation‐based genetic criteria should help provide better guidelines for genetic conservation of biodiversity (Hoban et al. 2020; Pérez‐Pereira et al. 2022).

## 4 Conclusion

(Waples 2022) wrote that “grokking *N*_*e*_: (truly understanding *N*_*e*_ deeply and intuitively)” is challenging. The analogy between *N*_*c*_ as a richness measure (the number of parents) and *N*_*e*_ as its equivalent diversity (the effective number of parents) helps to navigate the concept of an effective population size at a more intuitive level, as we are already used to thinking in terms of allelic richness, gene diversity and effective numbers of alleles. It also shows there is a clear theoretical and empirical foundation why we need to account for *N*_*c*_ when modeling populations genetically. Firstly, the pivotal role of *N*_*c*_ is predicted by the DPT (Jost 2010). Secondly, a large body of well‐established population genetic theory shows that *N*_*c*_ has a non‐negligible influence on evolutionary trajectories, even neutral ones. Thirdly, major advances in understanding neutral and adaptive diversity patterns, such as the relation between fecundity and genome‐wide genetic diversity (Romiguier et al. 2014), are directly derived from *N*_*c*_ and *N*_max_.

From simple snippets of established population genetic theory, we can logically deduce that *N*_*c*_ plays an essential role in the evolutionary trajectories of populations. After all, individuals in all their appearances and life stages are the subjects of chance and fortune, selection, mutation, and migration, not a theoretical equivalent number we call *N*_*e*_. *N*_*c*_ affects every aspect of population genetic processes, and influences directly and indirectly the interaction between them. We need to take variation in *N*_*c*_ into account next to *N*_*e*_ if we want to understand how evolution works and conserve biodiversity sustainably.

## Conflicts of Interest

Joachim Mergeay is an Editorial Board member of *Evolutionary Applications* and author of this article. To minimize bias, he was excluded from all editorial decision‐making related to the acceptance of this article for publication.

## Data Availability

The author has nothing to report.
